# Can radiomics be used to detect hypoxic–ischemic encephalopathy in neonates without magnetic resonance imaging abnormalities?

**DOI:** 10.1007/s00247-023-05680-z

**Published:** 2023-05-15

**Authors:** Xiamei Zhuang, Ke Jin, Huashan Lin, Junwei Li, Yan Yin, Xiao Dong

**Affiliations:** 1 Department of Radiology, Hunan Children’s Hospital, 86 Ziyuan Road, Yuhua District, Changsha, 410007 China; 2 Department of Pharmaceutical Diagnosis, GE Healthcare, Changsha, 410005 China

**Keywords:** Brain, Hypoxic–ischemic encephalopathy, Neonate, Magnetic resonance imaging, Radiomics

## Abstract

**Background:**

No study has assessed normal magnetic resonance imaging (MRI) findings to predict potential brain injury in neonates with hypoxic–ischemic encephalopathy (HIE).

**Objective:**

We aimed to evaluate the efficacy of MRI-based radiomics models of the basal ganglia, thalami and deep medullary veins to differentiate between HIE and the absence of MRI abnormalities in neonates.

**Materials and methods:**

In this study, we included 38  full-term neonates with HIE and normal MRI findings and 89 normal neonates. Radiomics features were extracted from T1-weighted images, T2-weighted images, diffusion-weighted imaging and susceptibility-weighted imaging (SWI). The different models were evaluated using receiver operating characteristic curve analysis. Clinical utility was evaluated using decision curve analysis.

**Results:**

The SWI model exhibited the best performance among the seven single-sequence models. For the training and validation cohorts, the area under the curves (AUCs) of the SWI model were 1.00 and 0.98, respectively. The combined nomogram model incorporating SWI Rad-scores and independent predictors of clinical characteristics was not able to distinguish HIE in patients without MRI abnormalities from the control group (AUC, 1.00). A high degree of fitting and favorable clinical utility was detected using the calibration curve with the Hosmer−Lemeshow test. Decision curve analysis was used for the SWI, clinical and combined nomogram models. The decision curve showed that the SWI and combined nomogram models had better predictive performance than the clinical model.

**Conclusions:**

HIE can be detected in patients without MRI abnormalities using an MRI-based radiomics model. The SWI model performed better than the other models.

**Graphical Abstract:**

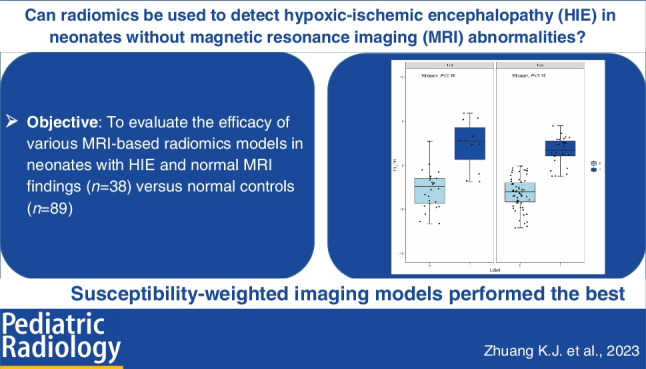

**Supplementary Information:**

The online version contains supplementary material available at 10.1007/s00247-023-05680-z.

## Introduction

Hypoxic–ischemic encephalopathy (HIE) is an important form of neonatal encephalopathy, has high mortality and morbidity and occurs in 1–8 cases per 1,000 live births [1]. The effects of HIE on the brain can lead to serious conditions, rapid progression and poor prognoses. However, patients experience varying degrees of neurological sequelae. The optimal method for evaluating suspected HIE is magnetic resonance imaging (MRI) [2]. Owing to the different examination times, injury patterns and protocols, some HIE patients may have normal or pseudonormal MRI findings [3]. When HIE occurs with brain injury, it is particularly challenging to identify and define. Furthermore, it has been reported that neurologic deficits may also occur in some neonates with normal brain MRI findings [4].

The Sarnat Grading Scale assesses the severity of HIE [5]. Normal or pseudonormal MRI findings can affect the Sarnat Score and most findings are classified as mild or moderate HIE. According to the Sarnat Grading Scale, infants with mild HIE were previously considered to have a good prognosis without long-term disability. Recent data analysis suggests an increased risk of behavioral dysregulation and attention deficit among school-aged children with a history of HIE at birth, even those who had mild HIE [6–8]. The prevalence of abnormalities in infants with mild HIE or normal brain MRI findings ranges from 20−60% [9]. Infants with mild HIE or HIE and normal brain MRI findings who were cooled had a lower incidence of brain injury than non-cooled infants [10]. Therefore, it is necessary to identify and treat HIE in infants with normal brain MRI findings.

Although conventional MRI is widely used in neonatal HIE, it provides limited information via human observation alone. Radiomics refers to the high-throughput computational extraction and analysis of features of digital medical images and the conversion of the information into mineable data [11]. These data can be subsequently analyzed to construct biomarkers for disease prediction and diagnosis by using feature selection. Radiomics can provide potentially valuable information beyond the limitations of human analysis [12]. The clinical applicability of radiomics has been investigated in several studies. Previous studies have shown the value of radiomics features in predicting disease, treatment response and prediction and prognosis in various cancers [13–17]. However, only a few studies have used texture analysis to evaluate ischemic changes in neonates. No study has assessed normal MRI findings in neonates with HIE to predict potential brain injury. The current study aimed to develop and validate radiomics models to differentiate brain injury from normal MRI findings in neonates with HIE.

## Materials and methods

### Patients and data collection

This retrospective study was approved by the Medical Ethics Committee of the Hunan Children’s Hospital of South China University. Owing to the retrospective analysis of anonymized data, the need for informed consent was waived.

The database of the Neonatology Department was reviewed to identify neonates with perinatal asphyxia and HIE between January, 2018 and April, 2022. The inclusion criteria were as follows: (1) neonates aged ≥37 weeks of gestation who underwent MRI; (2) normal MRI findings; and (3) available demographic, clinical and laboratory data. The exclusion criteria were as follows: (1) premature infants (gestational age <37 weeks), (2) infants with motion artifacts on MRI and (3) infants with metabolic diseases. A total of 38 patients who met the above criteria were included in the positive group.

**Fig. 1:**
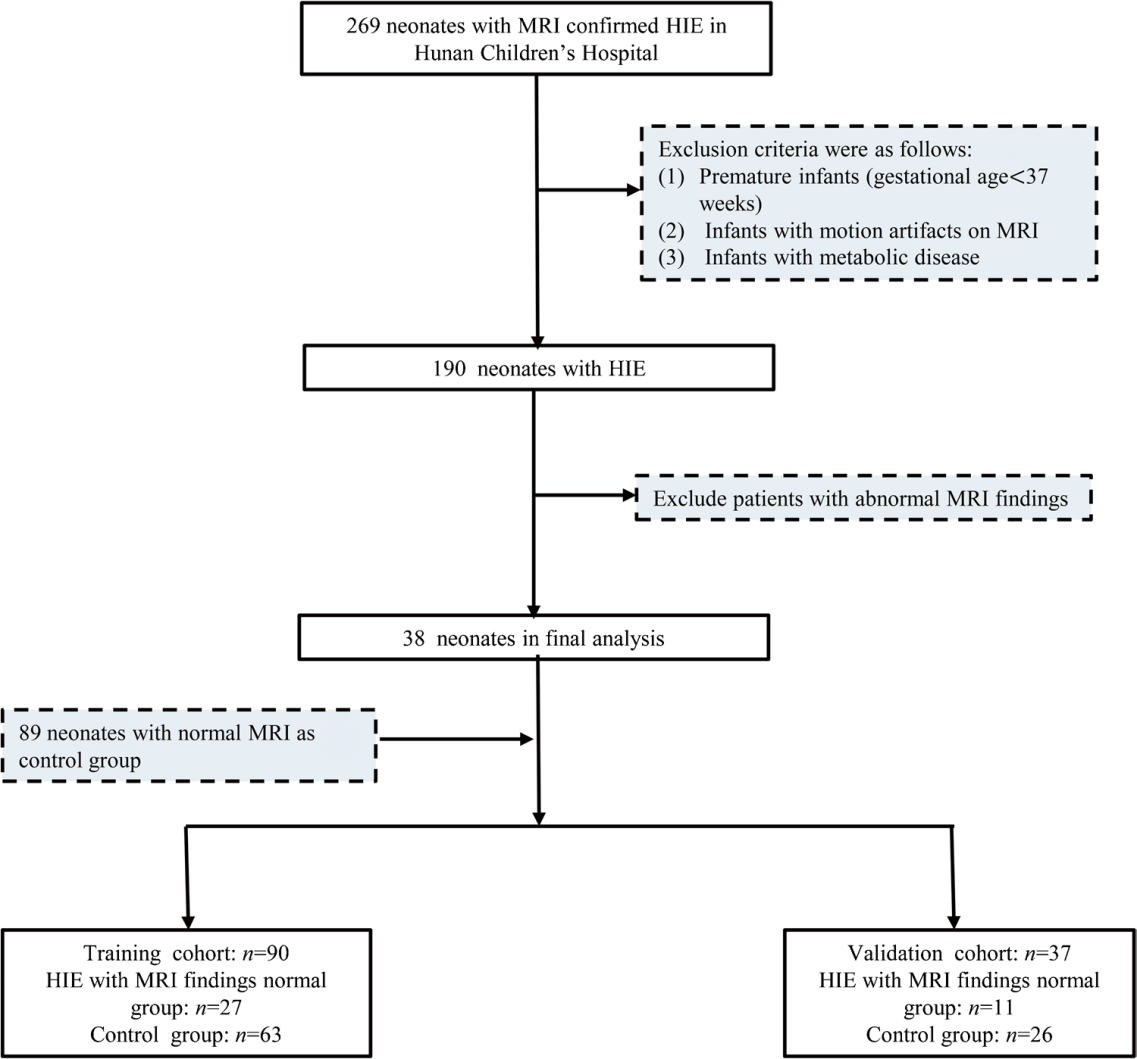
Flow chart summarizing enrolment of the study population. *HIE* hypoxic–ischemic encephalopathy, *MRI* magnetic resonance imaging

The control group consisted of neonates who underwent brain MRI within the first 2 weeks of life to investigate the possibility of a congenital central nervous system malformation. Infants without abnormalities observed on brain MRI were included. Based on the consensus of radiologists, the control group included 89 neonates with normal brain MRI findings. The identification and selection of the study cohort and the exclusion criteria are presented in Fig. 1.

### Magnetic resonance imaging acquisition and processing

All patients underwent brain MRI, including diffusion-weighted imaging and susceptibility-weighted imaging (SWI) scans. All brain MRI scans were performed at our hospital using a 3.0-tesla (T) MRI scanner (MAGNETOM Skyra, Siemens Healthcare, Erlangen, Germany or MAGNETOM Prisma, Siemens Healthcare, Erlangen, Germany) with an eight-channel head coil with the same MRI parameters. Additional details can be found in Supplementary Material 1.

MR images were preprocessed before segmentation and feature extraction to remove potential differences between the studies acquired from the two different scanners. The image preprocessing was performed by X.D., a pediatric radiologist with 5 years of experience. Additional details can be found in Supplementary Material 2.

### Model construction, evaluation and validation

MR images were moved to three-dimensional slicer software for segmentation and then saved for subsequent radiomics feature extraction. In this study, the deep medullary veins were assessed and quantified using the created regions of interest (ROIs) close to the lateral ventricles [18]. The ROI of the ganglia, thalami or deep medullary veins were manually determined by two pediatric radiologists (Y.Y. with 10 years of experience and X.D.) with unanimous agreement. The radiologists anonymized the clinical data. Additional details can be found in Supplementary Material 3.

Patients were randomly divided into two cohorts (training and validation cohorts) for each sequence in a ratio of 7:3. Considering that some features contribute to the positive classification performance and that others might add noise to it, the minimum redundancy maximum relevance (mRMR) was used to filter the features in our radiomics model to eliminate redundant and irrelevant features and retain the features with the maximum prediction efficiency. Thereafter, the least absolute shrinkage and selection operator (LASSO) was used to select effective and predictable features that are suitable for high-dimensional low-sample-size data with collinearity. A 10-fold cross-validation was used to select features with non-zero coefficients. After determining the number of features, the most predictive radiomics features were chosen to construct the radiomics model. The selected features in each model are termed the corresponding Rad-score. Prediction models based on radiomics feature parameters include the T1-weighted image (T1WI) basal ganglia model (T1WI-BG model), T1WI thalami model (T1WI-TH model), T2-weighted image (T2WI) basal ganglia model (T2WI-BG model), T2WI thalami model (T2WI-TH model), apparent diffusion coefficient (ADC) basal ganglia model (ADC-BG model), ADC thalami model (ADC-TH model) and SWI model. Based on the results of univariate and multivariate logistic regression analyses, the independent predictors of clinical characteristics were combined with the Rad-score obtained by the model that had the best performance to establish the combined nomogram model.

Clinical features from the univariate analysis (with statistical significance *P*<0.05) were used in the multivariate regression analysis. Features with *P*<0.05 in the multivariate regression analysis were included in the clinical model.

The diagnostic efficiency of different models was measured using receiver operating characteristic (ROC) curve and area under the curve (AUC) analyses in the training and validation cohorts. The Delong test was used to test the differences in the ROC curves. The predictive performances of the different models were calibrated and evaluated in the training and validation cohorts. The Hosmer−Lemeshow test was used to evaluate the calibration curves. Finally, decision curve analysis (DCA) was used to evaluate the clinical value of the different models.

All statistical analyses were performed using IBM Statistical Package for Social Sciences Statistics for Windows (Version 26.0; IBM Corp., Armonk, NY) and R software (Version 4.1.0; R Foundation for Statistical Computing, Vienna, Austria). Quantitative data were compared using Student’s t-test or the Wilcoxon test. Categorical data were compared using χ^2^ test. The “mRMRe” package was used to perform the mRMR analysis, and the “glmnet” package was used to execute the LASSO algorithm. The “pROC” package was used to plot the ROC curves. All statistical tests were two-sided, with statistical significance set at *P*<0.05.

## Results

### Clinical characteristics

A study group of 38 neonates with mild HIE and normal MRI findings were included. Based on the consensus of the radiologists, the control group included 89 neonates with normal brain MRI findings. In a ratio of 7:3, patients were randomly assigned to the training (*n*=90) or validation (*n*=37) cohorts. Details of the clinical characteristics and comparison between the HIE and control groups are presented in Table 1. Significant differences between the groups were found in some clinical manifestations (asphyxia, resuscitation, dyspnea and cyanosis), laboratory markers (alanine aminotransferase, aspartate aminotransferase, creatinine, creatine kinase isoenzyme, procalcitonin and lactic acid) and blood gas analysis (CO_2_, pH, and base excess). No variables were statistically different between the training and validation cohorts, thus suggesting reasonable classification.

**Table 1:** Demographic, clinical and laboratory features

Characteristic	Total (*n*=127)	HIE (*n*=38)	Control (*n*=89)	***P***	Training cohort (*n*=90)	Validation cohort (*n*=37)	*P*
Birth weight (mean $\pm$ sd)	3.3 $\pm$ 0.5	3.3 $\pm$ 0.5	3.3 $\pm$ 0.4	0.69	3.3 $\pm$ 0.4	3.3 $\pm$ 0.5	0.36
Head diameter (mean $\pm$ sd)	90.9 $\pm$ 3.9	90.6 $\pm$ 3.6	90.9 $\pm$ 4.1	0.69	90.8 $\pm$ 4.1	90.9 $\pm$ 3.4	0.88
Gestational time, week (mean $\pm$ sd)	39.2 $\pm$ 1.1	39.3 $\pm$ 0.9	39.1 $\pm$ 1.1	0.41	39.2 $\pm$ 1.1	39.20 $\pm$ 1.2	0.85
Age, day (mean $\pm$ sd)	7.4 $\pm$ 3.8	6.9 $\pm$ 2.1	8.1 $\pm$ 4.2	0.07	7.2 $\pm$ 4.3	7.8 $\pm$ 4.1	0.60
Asphyxia resuscitation, *n* (%)							
yes	37 (29.1%)	37 (97.4%)	89 (100%)		26 (28.9%)	11 (29.7%)	
no	90 (70.9%)	1 (2.6%)	0 (0%)	$<$ **0.01**	64 (71.1%)	26 (70.3%)	1.00
Dyspnea cyanosis, *n* (%)							
yes	50 (39.4%)	24 (63.2%)	26 (29.2%)		35 (38.9%)	15 (40.5%)	
no	77 (60.6%)	14 (36.8%)	63 (70.8%)	$<$ **0.01**	55 (61.1)	22 (59.5%)	1.00
Convulsions							
yes	18 (14.2%)	6 (15.8%)	12 (13.5%)		13 (14.4%)	5 (13.5%)	
no	109 (85.8%)	32 (84.2%)	77 (86.5%)	0.95	77 (85.6%)	32 (86.5%)	1.00
Sex, *n* (%)							
F	52 (40.9%)	11(28.9%)	41 (46.1%)		38 (42.2%)	14 (37.8%)	
M	75 (59.1%)	27 (71.1%)	48 (53.9%)	0.11	52 (57.8%)	23 (62.2%)	0.80
ALT (mean $\pm$ sd)	23.8 $\pm$ 35.4	35.8 $\pm$ 58.1	18.6 $\pm$ 16.8	**0.01**	25.5 $\pm$ 41.2	19.5 $\pm$ 12.3	0.38
AST (mean $\pm$ sd)	57.3 $\pm$ 44.2	86.7 $\pm$ 53.4	44.7 $\pm$ 32.7	$<$ **0.01**	59.4 $\pm$ 46.9	52.1 $\pm$ 36.9	0.40
Creatinine (mean $\pm$ sd)	45.5 $\pm$ 21.7	61.0 $\pm$ 20.7	38.9 $\pm$ 18.7	$<$ **0.01**	46.0 $\pm$ 21.9	44.3 $\pm$ 21.5	0.69
CK-MB (mean $\pm$ sd)	60.8 $\pm$ 80.5	100.0 $\pm$ 121.5	44.1 $\pm$ 46.2	$<$ **0.01**	65.0 $\pm$ 92.8	50.7 $\pm$ 35.7	0.36
Troponin (mean $\pm$ sd)	0.6 $\pm$ 6.0	1.9 $\pm$ 11.0	0.10 $\pm$ 0.0	0.12	0.8 $\pm$ 7.2	0.1 $\pm$ 0.1	0.52
Procalcitonin (mean $\pm$ sd)	2.8 $\pm$ 7.3	5.1 $\pm$ 10.2	1.8 $\pm$ 5.4	**0.01**	2.5 $\pm$ 6.7	3.5 $\pm$ 8.5	0.45
IL-6 (mean $\pm$ sd)	63.6 $\pm$ 169.9	55.0 $\pm$ 134.2	67.3 $\pm$ 183.7	0.71	62.0 $\pm$ 174.8	67.4 $\pm$ 159.6	0.87
Lactic acid (mean $\pm$ sd)	5.2 $\pm$ 1.7	5.7 $\pm$ 2.5	5.0 $\pm$ 1.2	**0.02**	5.3 $\pm$ 2.0	5.0 $\pm$ 1.0	0.38
D-dimer (mean $\pm$ sd)	2.5 $\pm$ 3.5	3.2 $\pm$ 2.9	2.2 $\pm$ 3.7	0.14	2.5 $\pm$ 3.8	2.4 $\pm$ 2.7	0.80
CO2 (mean $\pm$ sd)	36.1 $\pm$ 10.8	39.1 $\pm$ 10.8	34.8 $\pm$ 10.6	**0.04**	36.2 $\pm$ 11.2	36.0 $\pm$ 9.9	0.93
PO2 (mean $\pm$ sd)	73.3 $\pm$ 24.7	73.2 $\pm$ 25.3	73.3 $\pm$ 24.5	0.97	72.9 $\pm$ 24.7	74.3 $\pm$ 24.9	0.76
HCO3-ion (mean $\pm$ sd)	21.7 $\pm$ 5.5	21.2 $\pm$ 6.6	22.0 $\pm$ 4.9	0.45	21.7 $\pm$ 5.6	21.9 $\pm$ 5.2	0.86
pH (mean $\pm$ sd)	7.4 $\pm$ 0.1	7.3 $\pm$ 0.1	7.4 $\pm$ 0.1	$<$ **0.01**	7.4 $\pm$ 0.1	7.4 $\pm$ 0.1	0.82
BE (mean $\pm$ sd)	-2.6 $\pm$ 5.8	-4.6 $\pm$ 7.9	-1.7 $\pm$ 4.5	**0.01**	-2.5 $\pm$ 5.8	-2.6 $\pm$ 6.0	0.96

*ALT* alanine aminotransferase, *AST* aspartate aminotransferase, *BE* base excess, *CO2* carbon dioxide, *CK-MB* creatine kinase isoenzyme, *F* female, *HIE* hypoxic-ischemic encephalopathy, *IL-6* interleukin 6, *M* male, *PO2* partial pressure of oxygen

The bold values represent *P*-values $<$ 0.05 (statistical significance)

The multivariate regression analysis included all parameters with *P*<0.05 from the univariate analysis. The final results showed that creatinine and lactic acid levels were independent predictors of HIE (Table 2). A clinical model was established using independent predictors.

**Table 2:** Positive results of univariate and multivariate regression analysis of clinical characteristics

Univariate regression analysis				
Variable	Odds Ratio	Lower	Upper	*P*
Age	0.83	0.71	0.970	0.02
Dyspnea cyanosis	4.25	1.64	11.06	$<$ 0.01
ALT	1.03	1.00	1.06	0.03
AST	1.03	1.02	1.05	$<$ 0.01
Creatinine	1.05	1.03	1.08	$<$ 0.01
CK-MB	1.01	1.00	1.02	0.01
Procalcitonin	1.15	1.01	1.31	0.04
Lactic acid	1.35	1.04	1.76	0.03
pH	$<$ 0.01	$<$ 0.01	0.18	0.01
Multivariate regression analysis				
Variable	Odds Ratio	CI.95	*P*	
Creatinine	1.05	1.02–1.08	$<$ 0.01	
Lactic acid	1.37	1.04–1.81	0.02	

*ALT* alanine aminotransferase, *AST* aspartate aminotransferase, *CI .95 *95% confidence interval, *CK-MB* creatine kinase isoenzyme

### Radiomic feature selection and construction of the Rad-score

**Fig. 2:**
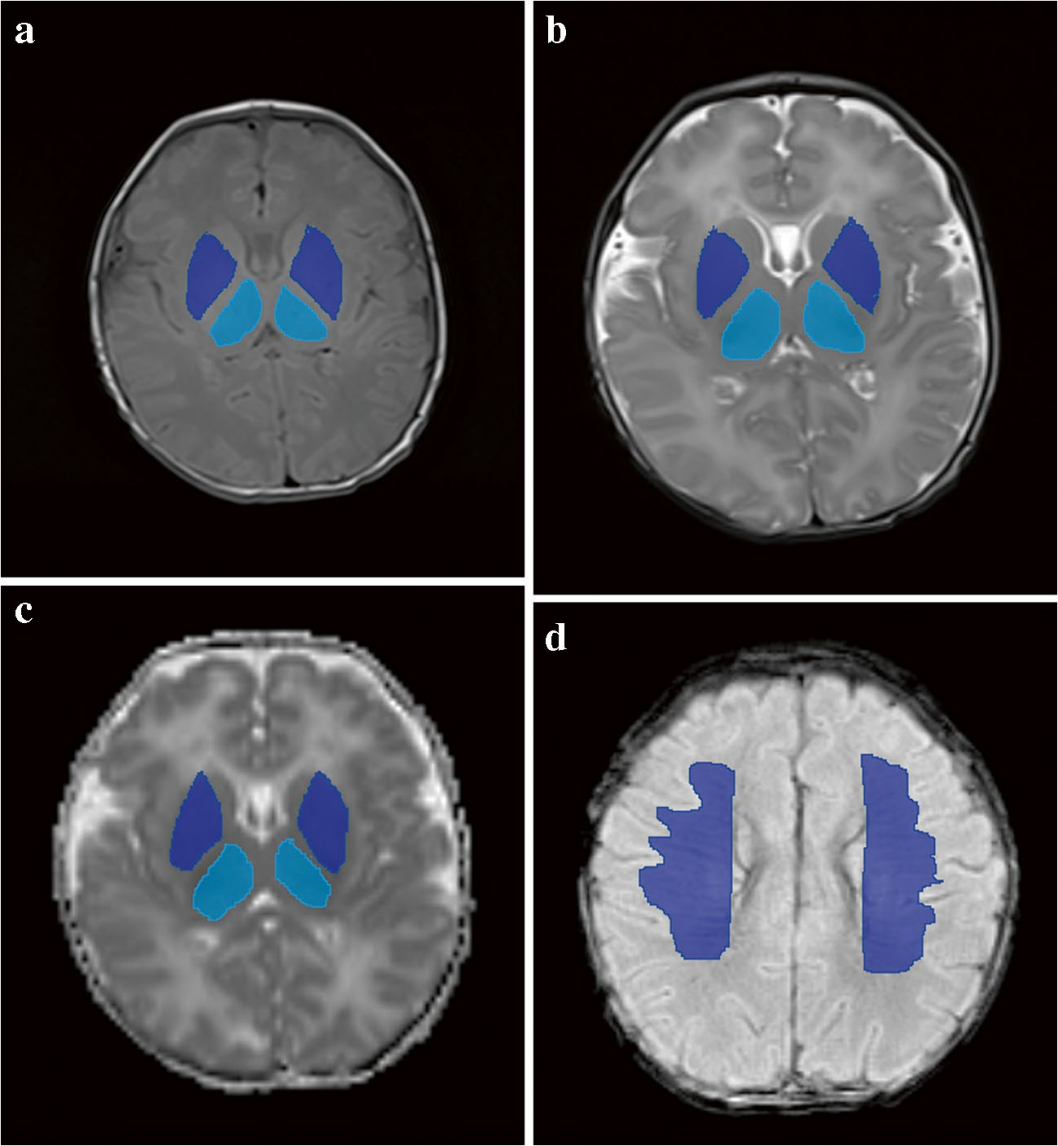
Radionics analysis on axial magnetic resonance images in a 37-week-old male neonate with mild hypoxic ischemic encephalopathy. **a**–**c** Regions of interest were placed on the basal ganglia (*dark blue*) and thalami (*light blue*) on the axial T1-weighted (W) images (**a**), T2-W images (**b**) and apparent diffusion maps (**c**). **d** Susceptibility-weighted image with regions of interest placed on the deep medullary veins (*dark blue*)

All radiomics features with non-zero coefficients in the LASSO logistic regression model were selected to build the differentiation model. After dimensionality reduction, the potential predictors were selected from the 1,316 features identified from the training cohort for each sequence, and the ROIs are shown in Fig. 2 and Supplementary Material 4. The equation for each Rad-score is presented in Supplementary Material 5. After screening the features extracted from the T1WI-BG, T1WI-TH, T2WI-BG, T2WI-TH, ADC-BG, ADC-TH and SWI models, a total of 7, 11, 7, 11, 8, 11 and 10 radiomics features were retained, respectively.

For the training and validation cohorts, the AUC of the SWI model were 1.00 and 0.98, respectively. Thus, the Rad-score obtained by the SWI combined with the independent predictors of clinical characteristics were used to establish the combined model (Supplementary Material 5).

### Performance and validation of different prediction models

**Fig. 3:**
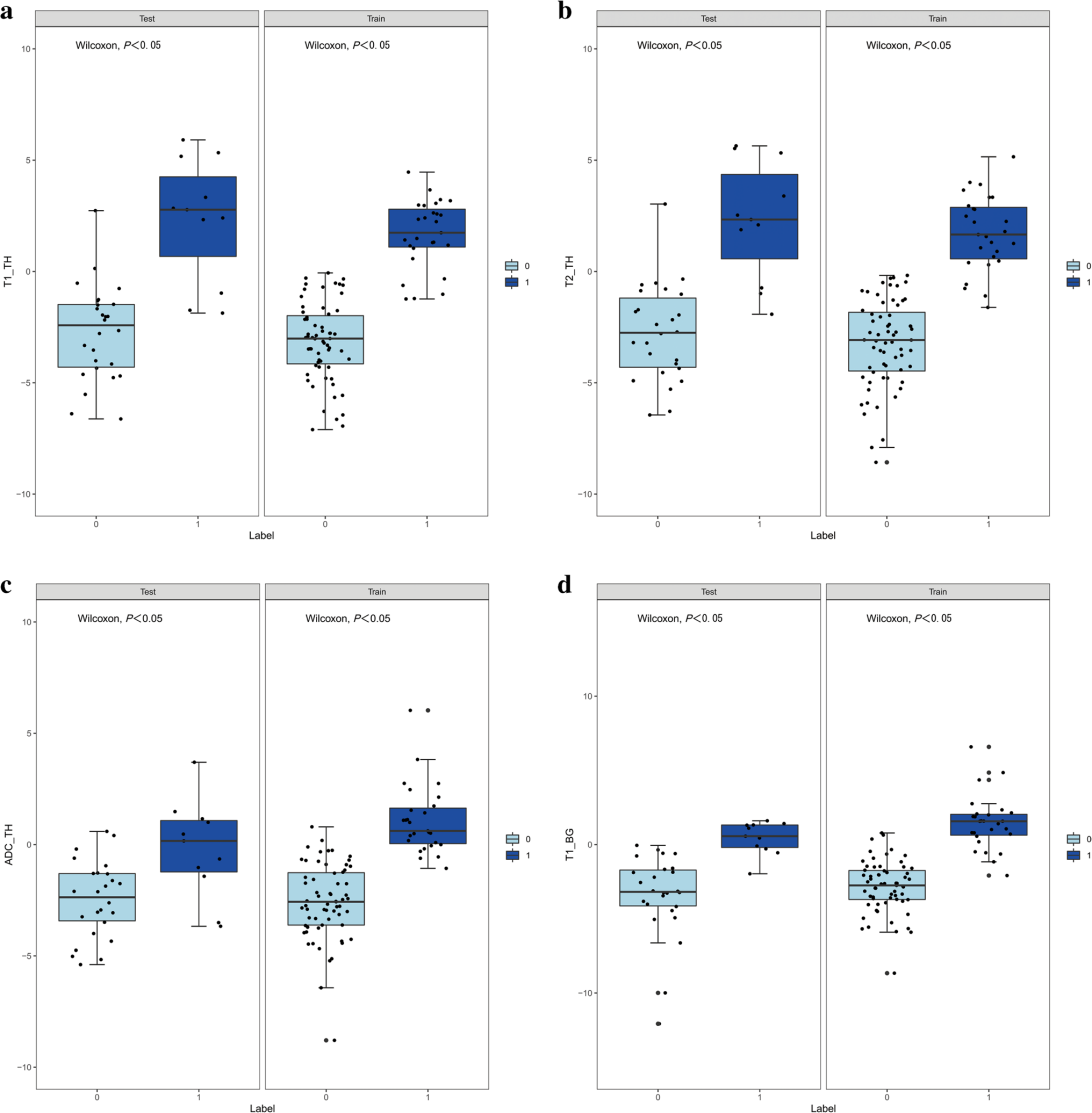

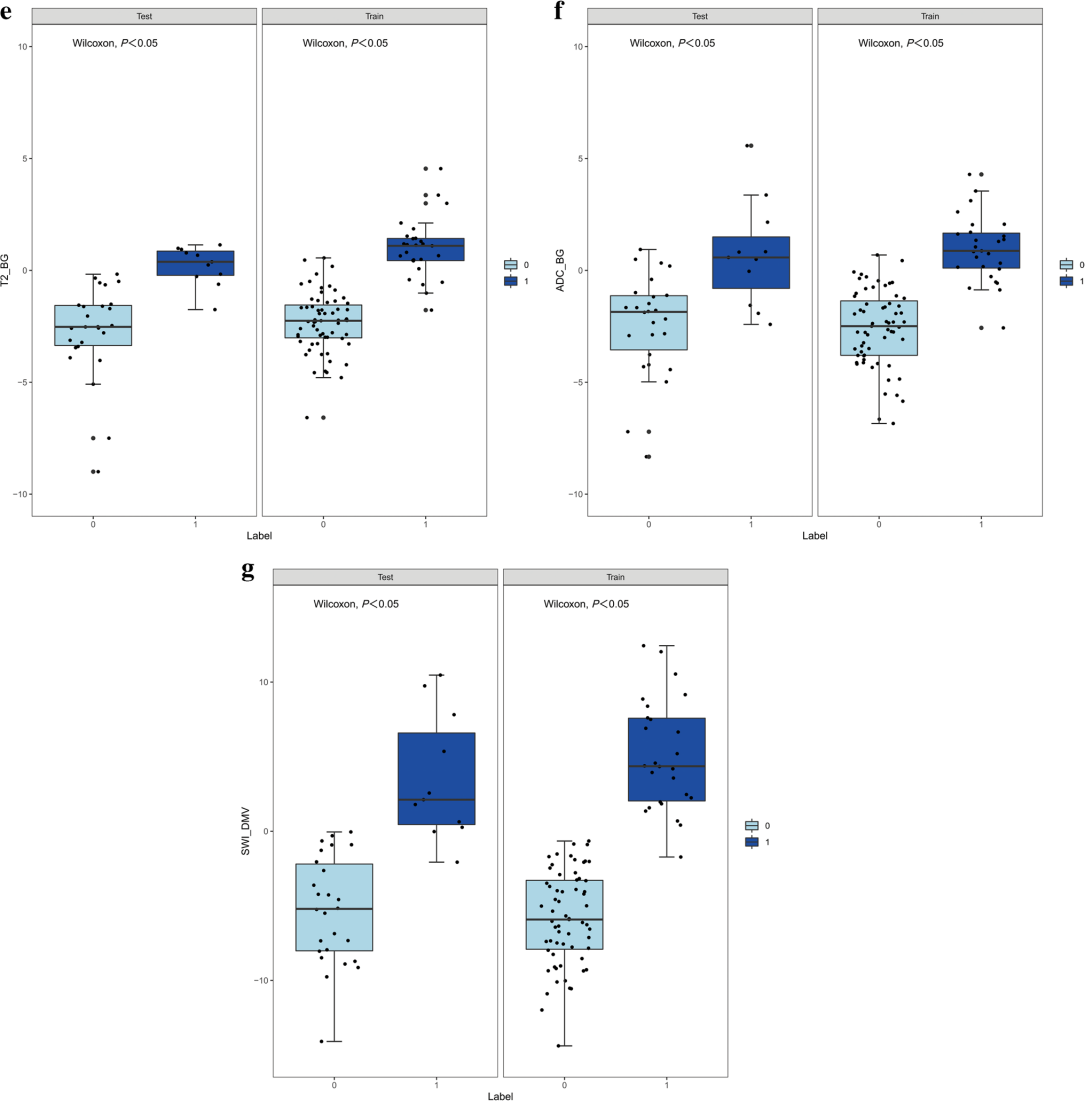
Rad-score scatterplots. **a**–**c** T1-weighted images (T1-W) (**a**), T2-weighted images (T2-W) (**b**) and apparent diffusion coefficient maps (**c**) with regions of interest placed on the thalami. **d**–**f** T1-W (**d**), T2-W (**e**) and apparent diffusion coefficient maps (**f**) with regions of interest placed on the basal ganglia. **g** Susceptibility-weighted imaging with regions of interest placed on the deep medullary veins. All plots show significantly higher Rad-scores in the hypoxic-ischemic encephalopathy with MRI findings normal group (*Label=1*) than in the control group (*Label=0*), in both the training cohort and the validation cohort

The Wilcoxon test was used to evaluate the difference between the two groups and the distribution of Rad-scores in the training and validation cohorts (Fig. 3). In the training cohort, HIE patients with normal brain MRI had a higher Rad-score than the control group in each MR sequence radiomics model and the combined models (*P*<0.05). This finding was confirmed in the validation cohort (*P*<0.05).

**Fig. 4:**
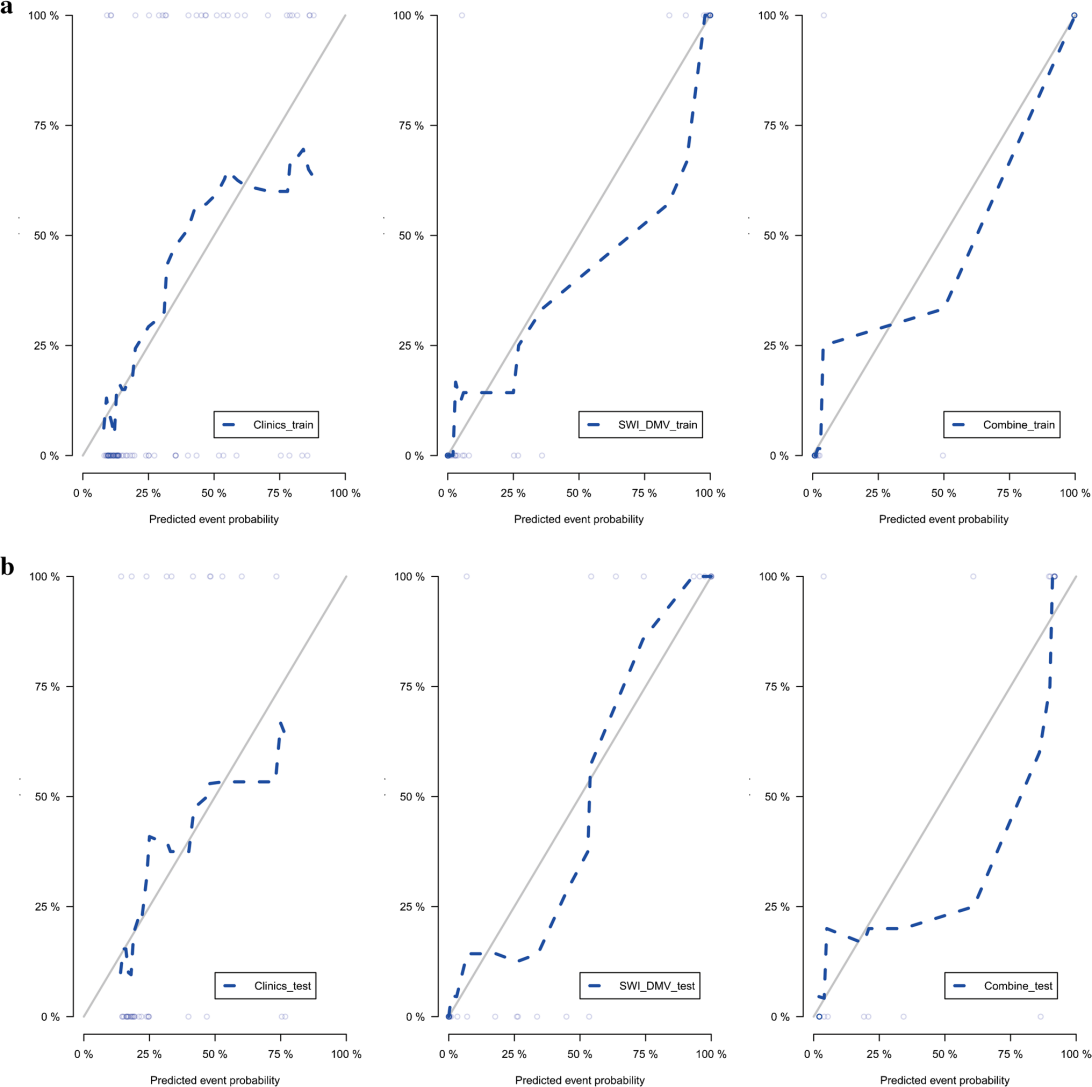
Calibration curves of the three models for the training (**a**) and validation (**b)** cohorts

The SWI model exhibited the best predictive performance among the seven single-sequence models. The AUCs of the training and validation cohorts in the SWI model were 1.00 (95% confidence interval [CI], 0.94−1.00) and 0.98 (95% CI, 0.82−0.99), respectively. In the training cohort, the AUCs of the T1WI-BG, T1WI-TH, T2WI-BG, T2WI-TH, ADC-BG, ADC-TH and clinical models, namely, 0.98 (95% CI, 0.85−0.97), 0.98 (95% CI, 0.79−0.94), 0.98 (95% CI, 0.83–0.96), 0.98 (95% CI, 0.89−0.99), 0.97 (95% CI, 0.77−0.92), 0.97 (95% CI, 0.79−0.94) and 0.82 (95% CI, 0.71−0.89), respectively, were relatively lower than the AUC of the SWI model. The AUC of the nomogram with creatinine, lactic acid and SWI Rad-score in the training cohort was 1.00 (95% CI, 0.94−1.00). The details are presented in Table 3 and Fig. 4.

**Table 3:** Accuracy and predictive value of different models

	AUC	Accuracy	95%CI	Sensitivity	Specificity	PPV	NPV
**Training cohort**							
T1WI_TH_model	0.98	0.88	0.79–0.94	1.00	0.83	0.71	1.00
T2WI_TH_model	0.98	0.96	0.89–0.99	0.85	1.00	1.00	0.94
ADC_TH_model	0.97	0.88	0.79–0.94	0.96	0.84	0.72	0.98
T1WI_BG_model	0.98	0.92	0.85–0.97	0.93	0.92	0.83	0.97
T2WI_BG_model	0.98	0.91	0.83–0.96	0.93	0.91	0.81	0.97
ADC_BG_model	0.97	0.86	0.77–0.92	0.96	0.81	0.68	0.98
SWI_DMV_model	1.00	0.99	0.94–1.00	0.96	1.00	1.00	0.98
Clinical model	0.82	0.81	0.71–0.89	0.85	0.79	0.64	0.93
Combine model	1.00	0.99	0.94–1.00	1.00	0.98	0.96	1.00
**Validation cohort**							
T1WI_TH_model	0.92	0.78	0.62–0.90	0.82	0.77	0.60	0.91
T2WI_TH_model	0.92	0.89	0.75–0.97	0.73	0.96	0.89	0.89
ADC_TH_model	0.79	0.81	0.65–0.92	0.64	0.89	0.70	0.85
T1WI_BG_model	0.92	0.87	0.71–0.96	0.91	0.85	0.71	0.96
T2WI_BG_model	0.92	0.84	0.68–0.94	0.91	0.81	0.67	0.96
ADC_BG_model	0.79	0.78	0.62–0.90	0.73	0.81	0.62	0.88
SWI_DMV_model	0.98	0.95	0.82–0.99	0.91	0.96	0.91	0.96
Clinical model	0.76	0.81	0.65–0.92	0.79	0.85	0.67	0.88
Combine model	0.98	0.84	0.68–0.94	0.91	0.81	0.67	0.95

*ADC* apparent diffusion coefficient, *AUC* area under the curve, *BG* basal ganglia, *CI* confidence interval, DM*V* deep medullary veins, *NPV* negative predictive value, *PPV* positive predictive value, *SWI* susceptibility-weighted imaging, *T1WI* T1-weighted image, *T2WI* T2-weighted image, *TH* thalami

**Fig. 5:**
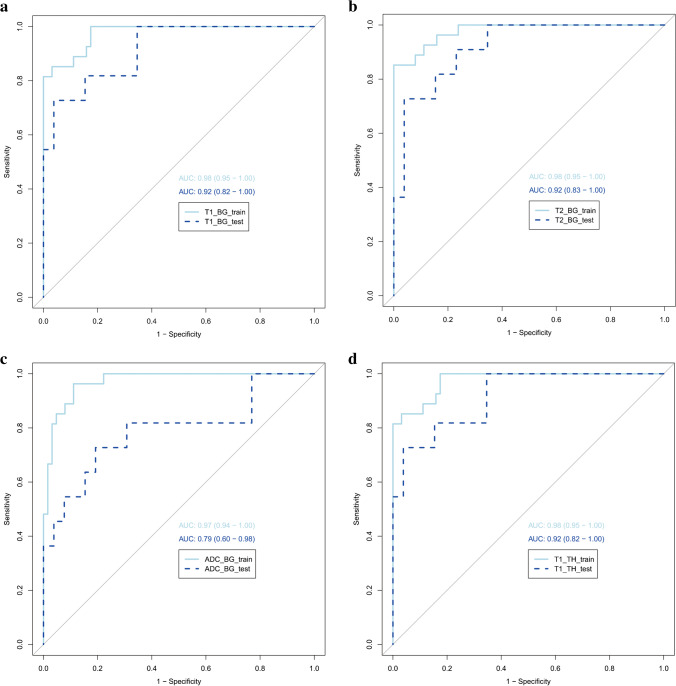

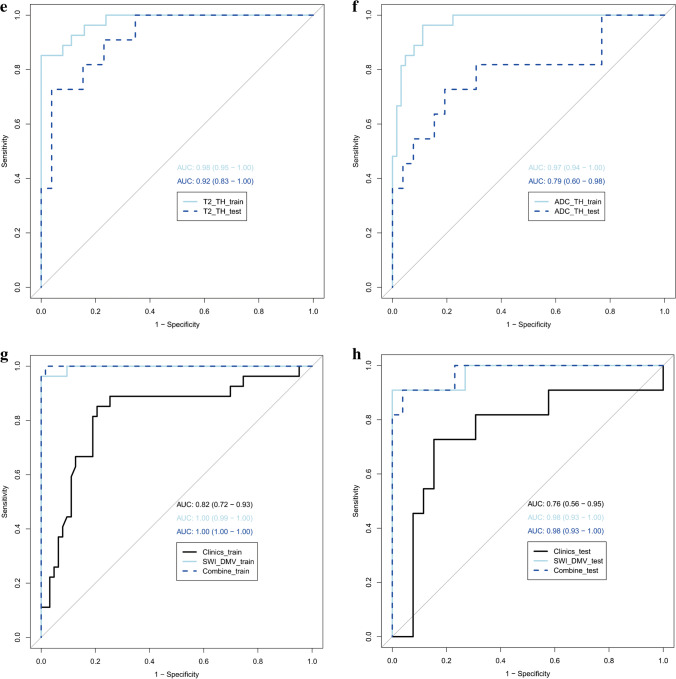
Receiver operating characteristic curves for the training and validation cohorts for different models. **a**–**c** T1-weighted images (T1-W) (**a**), T2-weighted images (T2-W) (**b**) and apparent diffusion coefficient (ADC) maps (**c**) with regions of interest placed on basal ganglia. **d**–**f** T1-W (**d**), T2-W (**e**) and ADC maps (**f**) with regions of interest placed on thalami. **g**, **h** Susceptibility-weighted imaging with regions of interest placed on deep medullary veins. The graphs represent the susceptibility-weighted model versus the clinical model versus the combined model for the training (**g**) and validation (**h**) cohorts. *ADC* apparent diffusion coefficient, *AUC* area under the curve, *BG* basal ganglia, *DMV* deep medullary veins, *SWI* susceptibility-weighted imaging, *TH* thalami

No significant differences were found between the ROC of the SWI model and the combined nomogram model in the training (*P*=0.38) and validation (*P*=1.00) cohorts. The calibration curve showed that the predicted probability of each model was in good agreement with the observed values (Fig. 5).

**Fig. 6:**
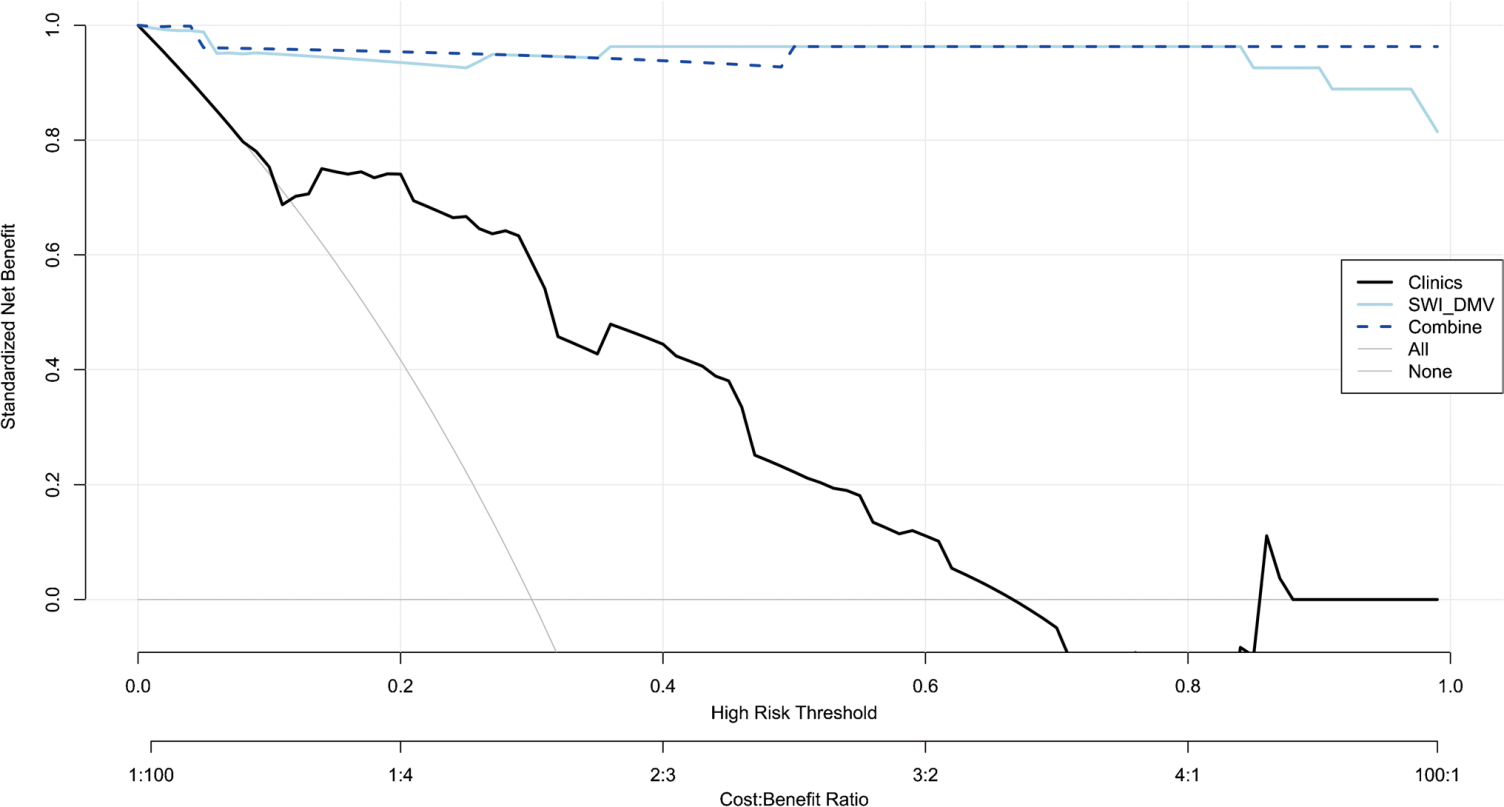
Decision curve analysis for the three models. The *light blue solid* line and *dark blue broken* line represent the susceptibility-weighted imaging radiomics model and combined model, respectively. The *black* line represents the clinical model. Decision curves showed that the susceptibility-weighted imaging radiomics and combined models achieved more clinical utility than the clinical model. *DMV* deep medullary veins, *SWI* susceptibility-weighted imaging

The DCA based on the clinical, SWI and combined nomogram models is shown in Fig. 6. The decision curve showed that the SWI and combined nomogram models had better predictive performance than the clinical model.

## Discussion

To the best of our knowledge, this is the first study to assess the utility of normal MRI in neonates with HIE to predict potential brain injury. MRI-based radiomics models of the basal ganglia, thalami and deep medullary veins allow for the accurate diagnosis of brain injury associated with HIE in neonates even when conventional brain MRI findings are normal. Our results show that radiomics features obtained from the basal ganglia and thalami on T1WIs, T2WIs and ADC maps have high diagnostic accuracy with AUC>0.90. The SWI model obtained from the deep medullary veins on the SWI had excellent diagnostic performance (AUC, 1.00), accuracy of 0.99, sensitivity of 0.96 and specificity of 1.00 in the training cohort. The combined nomogram model that was used together with the SWI Rad-score and creatinine and lactic acid levels did not significantly contribute to the differentiation between the brain injury with normal MRI findings in HIE and the normal groups, with an AUC of 1.00, an accuracy of 0.99, a sensitivity of 1.00 and a specificity of 0.98 in the training cohort. The Wilcoxon test, calibration curve and the Hosmer−Lemeshow test were performed to evaluate the predictive model. The results of our study suggest that the Rad-score value of each model is meaningful in the Wilcoxon test, thus indicating that radiomics is useful in each sequence and that radiomics features show the commonality of distinguishing the HIE group from the normal MRI and control groups in different sequences. There was good correlation between all models and the actual data.

Most studies have used conventional MRI features to predict brain injury in neonates with perinatal asphyxia. Conventional and further techniques for brain MRI have depicted the features of neonatal HIE, while Machie et al have used an MRI score to define abnormalities in HIE [19–21]. Parameters such as entropy, skewness and kurtosis, are commonly used. For example, Sarioglu et al. [22] used MRI-based texture features from the basal ganglia and thalami on apparent ADC maps and T1- and T2-WIs. The histogram entropy log-10 value was used as an indicator to differentiate between moderate-to-severe and mild HIE (*P*<0.001; odds ratio [OR], 266). An independent predictor of moderate-to-severe HIE was the absence of hyperintensity in the posterior limb of the internal capsule on T1WIs (*P*=0.012; OR, 17.11). Kim et al. [18] analyzed the value of the texture features of the deep medullary veins on SWI as a potential biomarker according to age and the presence of ischemic injury. Among these parameters, entropy showed a significant difference between the age groups (*P*=0.001). The ROC on skewness resulted in an AUC of 0.87 to differentiate infants with ischemic injury. The current study analyzed the relationship between traditional imaging signs and HIE brain injury even when MRI findings were normal. A series of quantitative imaging features can be extracted from the T1WI-BG, T1WI-TH, T2WI-BG, T2WI-TH, ADC-BG, ADC-TH and SWI models. Among the seven single-sequence models, the SWI model was superior to the other models in predicting potential brain injury with HIE in neonates without MRI abnormalities. The combined nomogram based on the SWI Rad-score and clinical factors may be used as a quantitative tool; however, it did not outperform the SWI model. According to the general pathophysiology, early pathological changes in HIE mainly include nerve cell degeneration, necrosis, brain edema, intracranial hemorrhage and cerebellar injury. After hypoxia occurs in brain tissue, cerebral blood flow perfusion decreases, arterioles show reactive dilation, oxygen intake by hypoxic brain tissue increases and hemodynamics at the microvascular level shows consequent damage, thus increasing the proportion of deoxyhemoglobin in venules. SWI is more sensitivite than conventional MRI in detecting abnormal venous dilatation in the brains of neonates with HIE [23]. Therefore, neonates with HIE have potential brain injuries even without MRI abnormalities. Radiomics refers to the high-throughput computational extraction and analysis of features from digital medical images and the conversion of information into mineable data. Wavelet_HHH_glszm (gray-level size-zone matrix [GLSZM]) _GrayLevelNonUniformity and wavelet_HLH_glcm (gray-level co-occurrence matrix [GLCM]) _maximal correlation coefficient were considered key parameters for the accuracy of the proposed SWI model according to their corresponding coefficients. The GLSZM consists of elements containing the number and size of gray areas. The gray-level band matrix includes features that describe the distribution of small/large areas and low/high gray areas. The GLCM is a matrix wherein the number of rows and columns represents the number of times the gray value is in a certain relationship (angle, distance), i.e. a second-order histogram. The features calculated on the GLCM included entropy, energy, contrast, homogeneity, dissimilarity and correlation [24–26]. The GLSZM and GLCM differ in their SWI sequence image gray level, image uniformity, contrast and homogeneity.

This study has several limitations. First, it is a single-center retrospective study with a lack of external verification, which might have led to case selection bias and limited generalizability. Second, although a relatively large number of neonates were included in this study, the cohort is small compared to those of other radiomics studies, particularly the HIE neonatal group with no MRI abnormalities; this might affect the general applicability of our results. A large-scale, prospective, multicenter study is required to validate our results. Third, manual segmentation was used to delineate the ROIs; automatic or semiautomatic segmentation, which are objective, were not used for comparison and verification. Fourth, various imaging protocols may potentially affect the radiomics. To handle this issue, image preprocessing before segmentation and feature extraction were performed to improve the robustness of the radiomic features. However, the variability of some imaging parameters that could not be normalized might have affected our results. The use of standardized imaging protocols is important to avoid low-quality unreliable results. Further research is needed to address these deficiencies.

## Conclusion

This study developed and compared eight models to assess the utility of normal MRI in neonates with HIE to predict potential brain injury. The results suggest that the SWI and combined nomogram models have potential for use in differentiating brain injury from normal MRI in HIE, with the SWI model offering the greatest diagnostic value.

## Supplementary Information

Below is the link to the electronic supplementary material.

Supplementary file1 (DOCX 914 KB)

## Data Availability

All data generated or analyzed during this study are included in this published article.
