# Supplementary material for: Can radiomics be used to detect hypoxic–ischemic encephalopathy in neonates without magnetic resonance imaging abnormalities?
Source: Pediatr Radiol. 2023 May 15;53(9):1927–40. doi: 10.1007/s00247-023-05680-z (PMC10421781; doi:10.1007/s00247-023-05680-z)
Supplement: Supplementary file 1 — Supplementary file1 (DOCX 914 KB) [file 247_2023_5680_MOESM1_ESM.docx]

**Supplementary material 1**

**Magnetic resonance imaging acquisition**

All patients underwent brain MRI scans, including diffusion weighted imaging (DWI) and susceptibility weighted imaging (SWI) scans. All brain MRI scans were performed at our hospital using a 3.0-tesla (T) MRI scanner (MAGNETOM Skyra, Siemens Healthcare, Erlangen, German or MAGNETOM Prisma, Siemens Healthcare, Erlangen, German) with an eight-channel head coil, using the same MR parameters. Axial T1-weighted image (T1WI), T2-weighted image (T2WI), apparent diffusion coefficient (ADC), and SWI extracted radiomics features. Parameters for T1WI were voxel size, 0.4 ×0.4 × 4.0 mm; slice thickness, 4.0 mm; TR, 800.0 ms; TE, 15.0 ms. Parameters for T2WI were voxel size, 0.6 × 0.6 × 4.0 mm; slice thickness, 4.0 mm; TR, 2230.0 ms; TE, 108.0 ms. The parameters for DWI were voxel size, 1.4 ×1.4 × 4.0 mm; slice thickness, 4.0 mm; TR, 5000.0 ms; TE, 60.0 ms. The parameters for SWI were field-of-view, 200 × 90.6 mm; voxel size, 0.8 ×0.8 × 2.0 mm; slice thickness, 2.0 mm; TR, 27.0 ms; TE, 20.0 ms. MR images of enrolled patients were exported in Digital Imaging and Communication in Medicine format in an institutional picture archiving and communication system and then converted to the NIFTI format using AK software (Artificial Intelligence Kit v.3.1.0. A, GE Healthcare, Shanghai, China).

**Supplementary material 2**

**Imaging processes**

We used AK software to perform the procedure. First, to eliminate the intrinsic dependency of the radiomic features on voxel size, a resampling method with a linear interpolation algorithm was used to normalize the voxel resolution. Higher-order texture analysis features were derived from different directions and scales, so the anisotropic voxels scanned at other sizes were resampled to form voxels, that is, 0.750 mm × 0.750 mm × 0.750 mm. Second, a Gaussian filter was used to remove “unwanted signals.” Because different scanners had the same gray level, gray-level normalization was not performed.

**Supplementary material 3**

**Image segmentation and radiomics feature extraction**

MR images were moved to 3D slicer software for segmentation and saved for subsequent radiomics feature extraction. In this study, deep medullary veins were assessed and quantified with the created regions of interests (ROI) close to the lateral ventricles. In the SWI sequences, ROIs were drawn at the right and left white matter ~~(WM)~~, involving the deep medullary veins. One axial slice was selected to draw the ROI at the level where the deep medullary veins showed a typical fan pattern of drainage into the subependymal vein. When drawing the ROI, we excluded subependymal veins and large cortical veins. The axial plane images on T1WI and T2WI sequences and ADC maps were exported for basal ganglia and thalami analysis. ROIs were drawn manually and bilaterally in each sequence on axial two-dimensional images of both the basal ganglia and thalami (Fig. 2).

We used AK software to extract radiomic features from each sequence. Four MR sequence images (T1WI, T2WI, ADC, and SWI) from 127 patients were included, and 1,316 features were extracted from each sequence. The features included first-order, shape, gray-level run-length matrix, gray-level co-occurrence matrix (GLCM), gray-level dependence matrix, gray-level size zone matrix (GLSZM), and neighborhood gray difference matrix. The selected image transformations were as follows: wavelet transformation (wavelet), level 1; logarithmic transformation (LoG), parameter sigma selection 2.0, 3.0; local binary mode (LBP), level 2, radius 1.0, subdivision select 1.

**Supplementary material 4**

**Supplementary figures 1-7** The least absolute shrinkage and selection operator (LASSO) including the selection of the regular parameter \(\lambda\) and determination of the number of features.

**Figure 1 ADC-BG model**

**
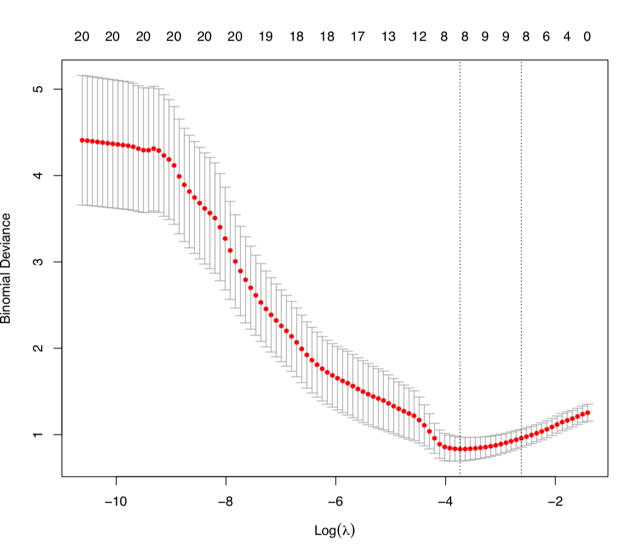
**
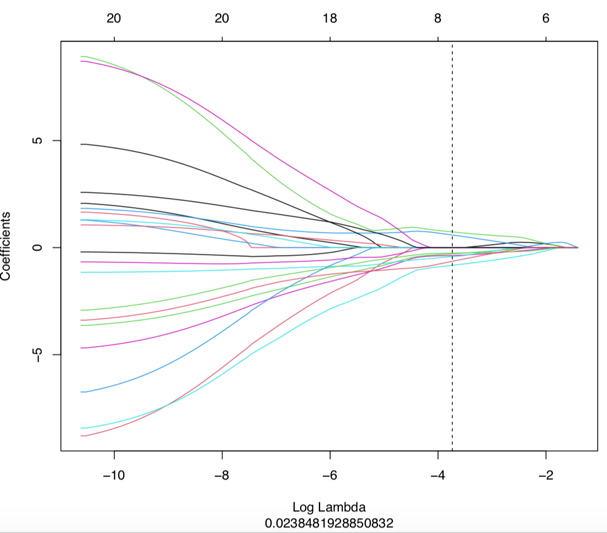


**Figure 2 ADC-TH model**

**
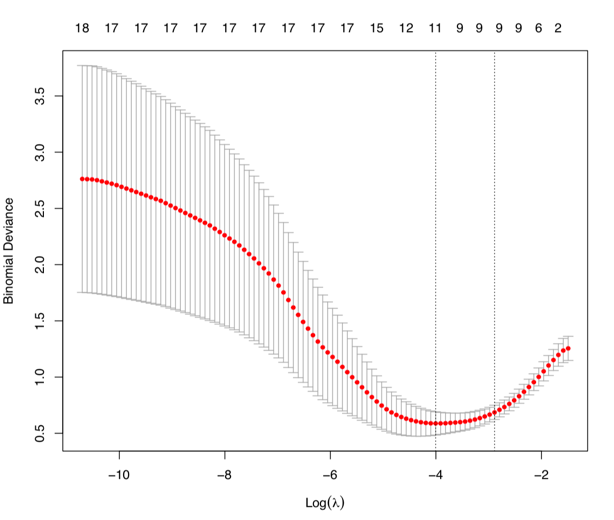

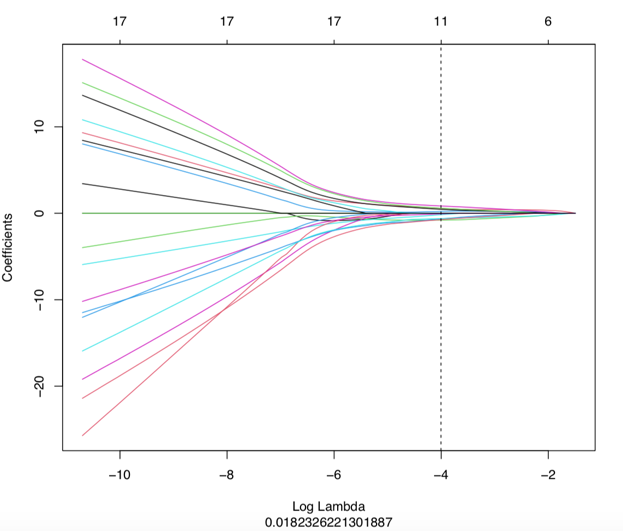
**

**Figure 3 SWI model**

**
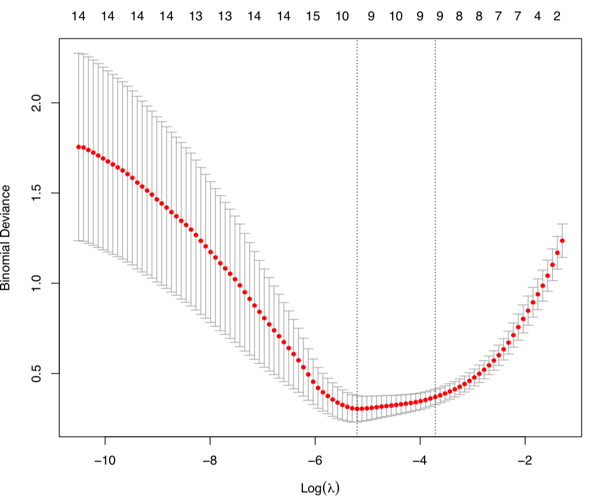

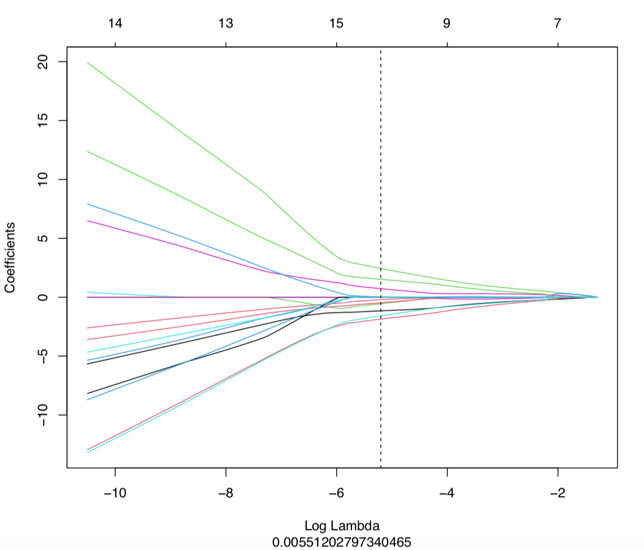
**

**Figure 4 T1-BG model**

**
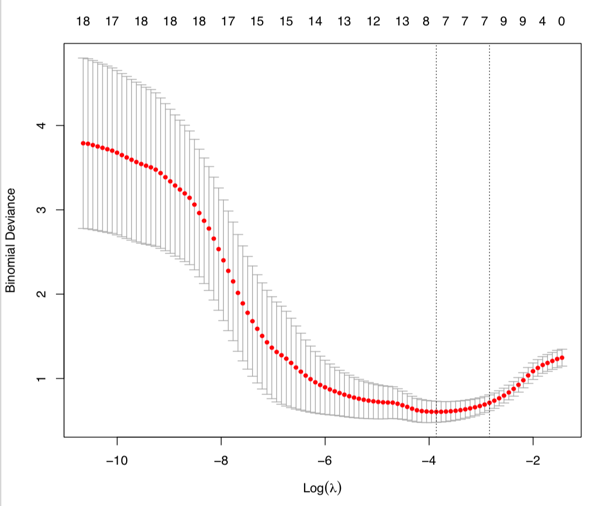

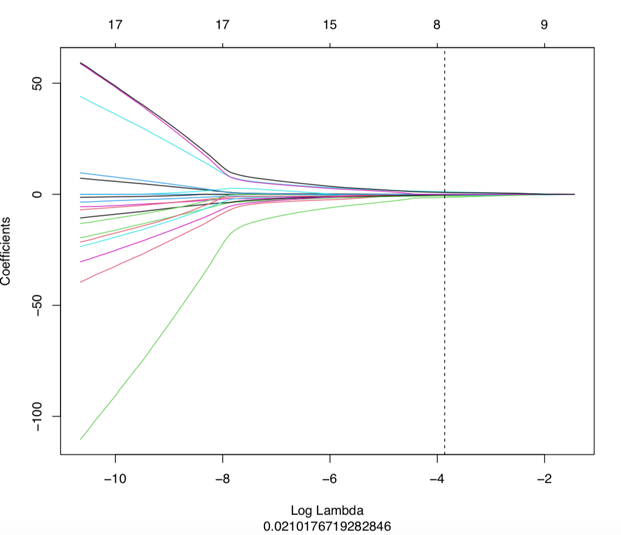
**

**Figure 5 T1-TH model**

**
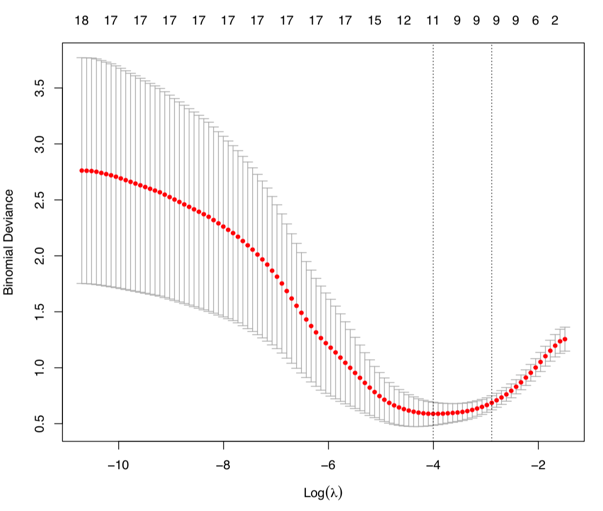

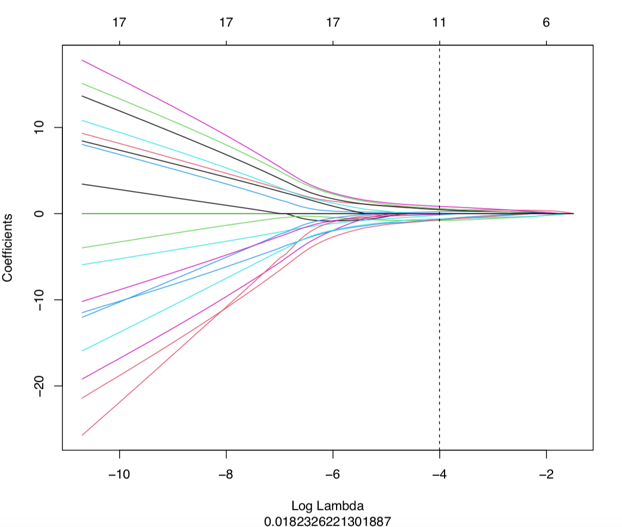
**

**Figure 6 T2-BG model**

**
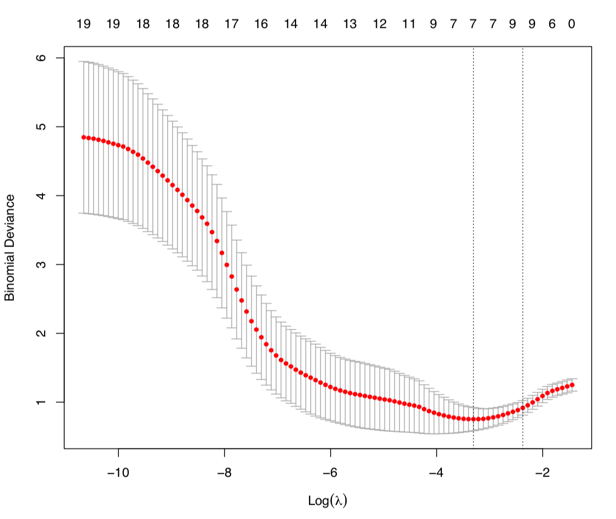

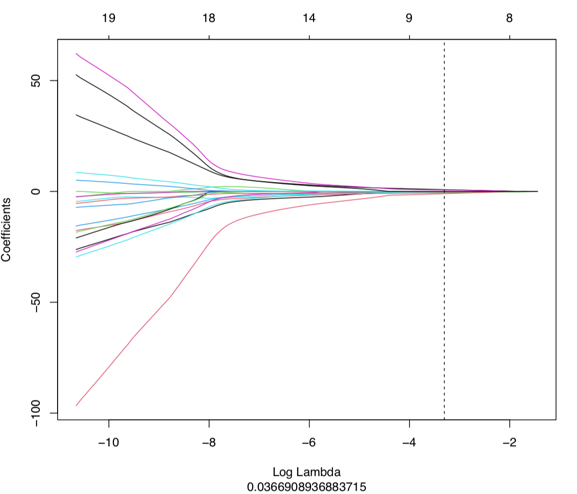
**

**Figure 7 T2-TH model**

**
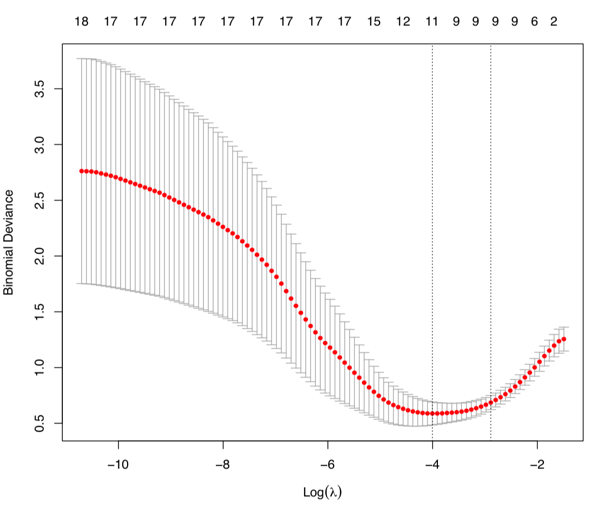

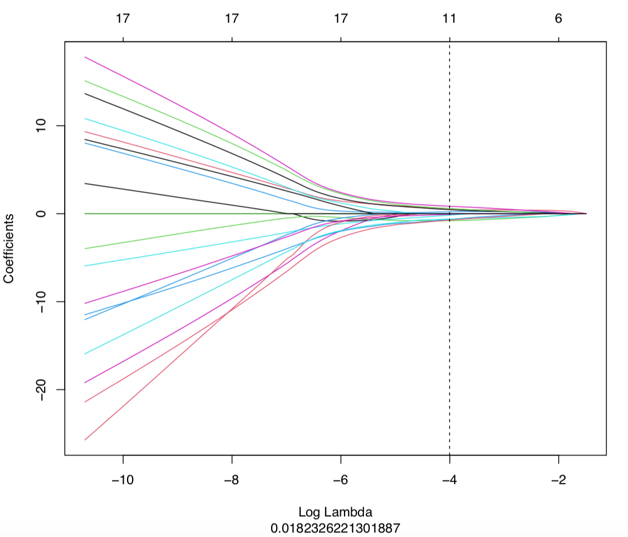
**

**Supplementary material 5**

**Equations**

**Equation 1** Rad-score formula for ADC-BG

> print(radscore)

[1] "radscore=0.735143423201127*wavelet_HLH_glszm_LargeAreaLowGrayLevelEmphasis+0.590348316093196*original_shape_Sphericity+-0.257339006756565*wavelet_LHH_glcm_Idmn+-0.290121221969974*wavelet_HLH_firstorder_Entropy+-0.367636061673566*original_shape_SurfaceArea+-0.420963916678219*wavelet_LLL_glszm_GrayLevelNonUniformity+-0.638780994323392*wavelet_LHH_glcm_MCC+-0.825235172480782*log_sigma_2_0_mm_3D_ngtdm_Complexity+-1.50877361923523*(Intercept)"

> sink()

**Equation 2** Rad-score formula for ADC-TH

> print(radscore)

[1] "radscore=0.842128430804047*original_shape_Maximum2DDiameterColumn+0.58807641208849*log_sigma_3_0_mm_3D_glszm_GrayLevelNonUniformity+0.575310119884333*wavelet_HLL_glszm_ZoneEntropy+0.459071878424364*log_sigma_3_0_mm_3D_firstorder_Maximum+0.214486768176421*wavelet_LLL_glrlm_HighGrayLevelRunEmphasis+-0.0671105027135498*log_sigma_2_0_mm_3D_glszm_SizeZoneNonUniformityNormalized+-0.167285902352168*wavelet_HHL_firstorder_Kurtosis+-0.612905674961401*wavelet_LHL_firstorder_Skewness+-0.64907773021111*wavelet_LLL_glszm_LowGrayLevelZoneEmphasis+-0.732540467958079*original_shape_MinorAxisLength+-0.791709834397514*original_shape_Maximum2DDiameterRow+-1.56565344534325*(Intercept)"

> sink()

**Equation 3** Rad-score formula for SWI

> print(radscore)

[1] "radscore=2.43804812771305*wavelet_HHH_glszm_GrayLevelNonUniformity+1.51972772371499*lbp_3D_k_gldm_SmallDependenceEmphasis+0.723928974807466*wavelet_HLH_glszm_ZoneEntropy+0.026170863542864*wavelet_HHH_gldm_DependenceEntropy+-0.217276691624361*lbp_3D_m1_firstorder_Median+-0.472210980106981*wavelet_HLH_glrlm_LongRunEmphasis+-0.555006911735788*wavelet_HLH_glszm_ZonePercentage+-1.15897859578284*wavelet_HHL_glrlm_LongRunEmphasis+-1.58311607309977*wavelet_HHH_glrlm_LongRunEmphasis+-1.85864513483325*wavelet_HLH_glcm_MCC+-2.51374636458375*(Intercept)"

> sink()

**Equation 4** Rad-score formula for T1-BG

> print(radscore)

[1] "radscore=1.11066105516041*lbp_3D_m1_firstorder_Minimum+0.807761879365246*original_gldm_DependenceEntropy+0.0223662807531044*log_sigma_2_0_mm_3D_firstorder_Energy+-0.301215806826013*lbp_3D_m1_firstorder_InterquartileRange+-0.343707267624223*log_sigma_3_0_mm_3D_firstorder_Kurtosis+-0.757782563280064*original_shape_Maximum2DDiameterColumn+-1.42512657693813*wavelet_LLL_firstorder_Kurtosis+-1.76619856290309*(Intercept)"

> sink()

**Equation 5** Rad-score formula for T1-TH

> print(radscore)

[1] "radscore=0.842128430804047*original_shape_Maximum2DDiameterColumn+0.58807641208849*log_sigma_3_0_mm_3D_glszm_GrayLevelNonUniformity+0.575310119884333*wavelet_HLL_glszm_ZoneEntropy+0.459071878424364*log_sigma_3_0_mm_3D_firstorder_Maximum+0.214486768176421*wavelet_LLL_glrlm_HighGrayLevelRunEmphasis+-0.0671105027135498*log_sigma_2_0_mm_3D_glszm_SizeZoneNonUniformityNormalized+-0.167285902352168*wavelet_HHL_firstorder_Kurtosis+-0.612905674961401*wavelet_LHL_firstorder_Skewness+-0.64907773021111*wavelet_LLL_glszm_LowGrayLevelZoneEmphasis+-0.732540467958079*original_shape_MinorAxisLength+-0.791709834397514*original_shape_Maximum2DDiameterRow+-1.56565344534325*(Intercept)"

> sink()

**Equation 6** Rad-score formula for T2-BG

> print(radscore)

[1] "radscore=0.808423683847909*lbp_3D_m1_firstorder_Minimum+0.669734882765873*original_gldm_DependenceEntropy+0.0432547656980808*log_sigma_2_0_mm_3D_firstorder_Energy+-0.152112995356579*lbp_3D_m1_firstorder_InterquartileRange+-0.335536527892039*log_sigma_3_0_mm_3D_firstorder_Kurtosis+-0.620382616708061*original_shape_Maximum2DDiameterColumn+-1.00417853091819*wavelet_LLL_firstorder_Kurtosis+-1.48954375718374*(Intercept)"

> sink()

**Equation 7** Rad-score formula for T2-TH

> print(radscore)

[1] "radscore=0.842128430804047*original_shape_Maximum2DDiameterColumn+0.58807641208849*log_sigma_3_0_mm_3D_glszm_GrayLevelNonUniformity+0.575310119884333*wavelet_HLL_glszm_ZoneEntropy+0.459071878424364*log_sigma_3_0_mm_3D_firstorder_Maximum+0.214486768176421*wavelet_LLL_glrlm_HighGrayLevelRunEmphasis+-0.0671105027135498*log_sigma_2_0_mm_3D_glszm_SizeZoneNonUniformityNormalized+-0.167285902352168*wavelet_HHL_firstorder_Kurtosis+-0.612905674961401*wavelet_LHL_firstorder_Skewness+-0.64907773021111*wavelet_LLL_glszm_LowGrayLevelZoneEmphasis+-0.732540467958079*original_shape_MinorAxisLength+-0.791709834397514*original_shape_Maximum2DDiameterRow+-1.56565344534325*(Intercept)"

> sink()

**Equation 8**

Nomoscore formula

[1] "nomoscore=-36.5807363815032*(Intercept)+3.05918302834458*Creatinine+-3.04247182025594*Lactic_acid+72.8720071490848*SWI_DMV"
